# Jiang Tang Xiao Ke Granule Play an Anti-diabetic Role in Diabetic Mice Pancreatic Tissue by Regulating the mRNAs and MicroRNAs Associated with PI3K-Akt Signaling Pathway

**DOI:** 10.3389/fphar.2017.00795

**Published:** 2017-11-01

**Authors:** Fang-Fang Mo, Tian An, Zi-Jian Zhang, Yu-Fei Liu, Hai-Xia Liu, Yan-Yun Pan, Jia-Nan Miao, Dan-Dan Zhao, Xiu-Yan Yang, Dong-Wei Zhang, Guang-Jian Jiang, Si-Hua Gao

**Affiliations:** ^1^Diabetes Research Center, Beijing University of Chinese Medicine, Beijing, China; ^2^Department of Biomedical Sciences, Paul L. Foster School of Medicine, Texas Tech University Health Sciences Center El Paso, Texas, TX, United States; ^3^Beijing University of Chinese Medicine Third Affiliated Hospital, Beijing, China

**Keywords:** Jiang Tang Xiao Ke Granule, KK-Ay diabetic mice, Pancreas tissue, microRNA, INS-1 cell

## Abstract

**Purpose:** To investigate the effect of JTXK granule on the expression pattern of miRNA in pancreatic tissue of KKAy diabetic mice, and to explore the molecular mechanism and pathways of JTXK granule in anti-diabetic effect.

**Methods:** We used high fat diet (HFD) to induce the KKAy diabetic mice and screened the differentially expressed miRNAs (DEMs) between JTXK-treated group (*n* = 6) and the diabetic group (*n* = 6) using MicroRNA (miRNA) Microarray. C57BL/6J mice were given a normal diet as the control group (*n* = 6). Subsequently, miRNA target gene prediction, GO and Pathway analysis were used to explore the function of DEMs. Finally, the mechanism of anti-diabetic effects of JTXK granule was tested by *in vitro* INS-1 pancreatic β-cell experiment.

**Results:** The blood glucose and body weight of JTXK-treated group was significantly lower compared with the model group. Moreover, a total of 45 miRNAs with significant differences were detected in the model group and the JTXK-treated group (*P* ≤ 0.05, Fold Change > 2). Further, miRNA-mRNA analysis showed that the differential expression of mmu-miR-192-5p, mmu-miR-291a-3p, mmu-miR-320-3p, mmu-miR-139-5p and mmu-miR-378a-3p are closely related to pancreatic histological changes. In addition, pathway analysis showed that the DEMs were closely related to PI3K-Akt Signaling Pathway. Furthermore, the levels of serine/threonine-protein kinase (Akt), phosphorylated Akt (p-Akt) and phosphorylated forkhead transcription factor O1 (p-Foxo1) in INS-1-FOXO1 overexpressing model cells were lower than those in normal group, while JTXK granules could increase the expression of Akt, p-Akt and p-Foxo1.

**Conclusions:** The results showed that JTXK granule could play an anti-diabetic role by regulating the mRNA and miRNAs associated with PI3K-Akt pathway in diabetic mice pancreatic tissue.

## Introduction

Hyperglycemia, insulin resistance (IR), and impaired β-cell function constitute the pathologic basis of type two diabetes mellitus (Kahn et al., 2007; Gao et al., 2016). PI3K-Akt signaling pathway not only reduce insulin resistance, but also promote the uptake of glucose in peripheral target tissue, and plays an important role in pancreatic β-cell secretion of insulin (Shoelson et al., 2006; Wang et al., 2015). Akt and PI3K have a significant influences in insulin transduction (Yu et al., 2017). IR can reduce the tyrosine phosphorylation level of the insulin receptor substrate (IRS), thereby interfering with the activation of PI3K and Akt, leading to abnormal expression of insulin PI3K-Akt signaling pathway (Taniguchi et al., 2006; Granata et al., 2007). Foxo1, a downstream transcription factor activated by PI3K-Akt pathway, involved in cellular energy metabolism, cell apoptosis, and insulin signaling (Ponugoti et al., 2012). Foxo1 abnormal expression has an important relationship with IR, so the inhibition of abnormal expression of Foxo1may be a new target in the treatment of diabetes mellitus.

MicroRNAs, a short non-coding regulatory RNA molecules, has become an important participant in gene regulation and has a significant impact on the diabetes process (Dlouhá and Hubáček, 2017; Ofori et al., 2017). Studies have shown that miRNAs are a key regulator of lipid and glucose metabolism and play a pivotal role in the pathogenesis of metabolic diseases by affecting the states and functions of liver, adipose tissue and pancreatic (Iacomino and Siani, 2017). Additionally, many miRNAs are reported to be involved in pancreatic development and play a post-transcriptional regulatory role in insulin secretion and beta-cell differentiation transcripts in IR individuals (Kaviani et al., 2016; Bai et al., 2017; Massart et al., 2017; Sebastiani et al., 2017). In addition, Studies have suggested that miRNAs can regulate multiple signaling pathways or network associated with cellular processes by regulating multiple target gene expression (Wilczynska and Bushell, 2015). Therefore, miRNAs is expected to become a new treatment for diabetes as a therapeutic target.

JTXK granule is a pure Chinese medicine granules, including Rehmannia (DiHuang), Pueraria (Gegen), Fructuscorni (ShanYuRou), Ginseng (RenShen), Radix salviae miltiorrhizae (DanShen), and other five Chinese herbs in accordance with a certain proportion of composition. In previous studies we found JTXK granule can improving liver lipid metabolism in T2DM mice and inhibiting apoptosis in INS-1 cells (Rui et al., 2016; Zhang et al., 2016b). At the same time JTXK granules can also increase the expression of glucose transporter 4 (Glut4), phosphatidylinositol 3-kinase (PI3K), and insulin receptorsubstrate-1 (IRS-1) in skeletal muscle of diabetic mice at gene and protein levels (Na et al., 2016). In addition, JTXK granule can reduce lipid and glucose levels in both diabetes mice and rats by preventing islet damage and implementing antioxidant effects (Zhao et al., 2014; Zhang et al., 2016a). Therefore, it is important to find mRNA, miRNA, and pathways related to the anti-diabetic effect of JTXK granules in pancreatic tissue, which not only conducive to the disclosure of pharmacological mechanisms but also contribute to explore the molecular targets in the anti-diabetic effect of JTXK granule.

## Materials and methods

### Preparation of JTXK granule

**Figure 1:**
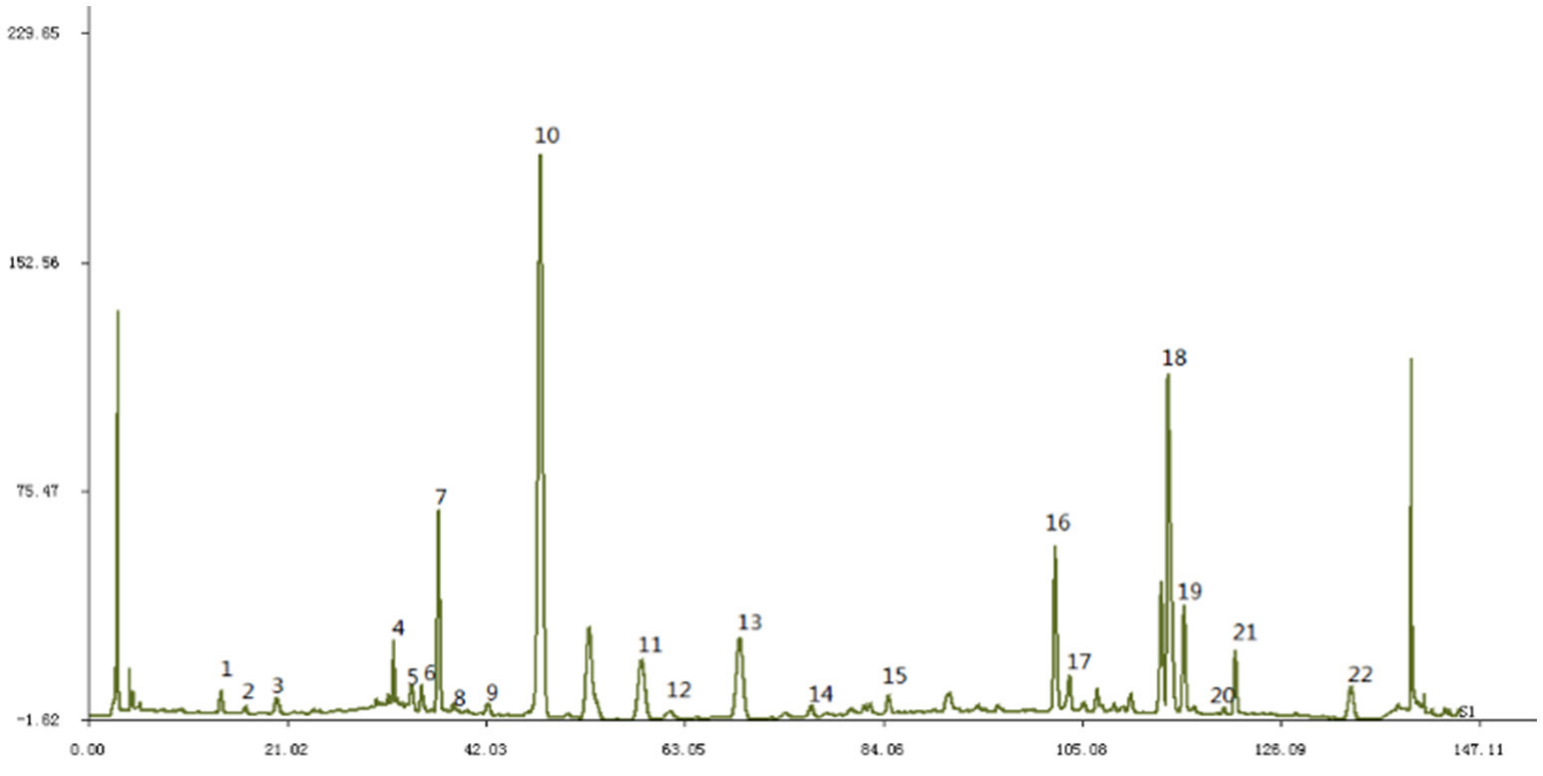
The fingerprint of JTXK granule. Paeonol (22), Sal B (18), Berberine (19), Coptisine (16), Puerarin (10).

JTXK granule were prepared as previously described (Yu et al., 2017). Briefly, the original herbs of JTXK granule [Rehmannia (DiHuang), Pueraria (Gegen), Fructuscorni (ShanYuRou), Ginseng (RenShen), Radix salviae miltiorrhizae (DanShen) etc.] were purchased from Beijing Tongrentang Pharmacy, and the authenticity of these herbs were verified by Professor Chunsheng Liu (School of Chinese Materia Medica, Beijing University of Chinese Medicine). JTXK granule was made from the ethanoic extracts and pooled aqueous and the final yield of 20% (w/w; i.e., every 1 g of extract was obtained from 5 g of herbs) was obtained, then placed at 4°C for later use. Finally, the main components of JTXK granule were obtained by the High Performance Liquid Chromatography (HPLC) fingerprint system (Figure 1).

### Animal treatment

KKAy and C57BL/6J mice used in this study were provided by Beijing Hua Fu Kang Bioscience Co. Ltd. (Beijing, China). 8-week-old male KKAy mice were fed with HFD (67.3% standard chow, 20% sucrose, 10% lard, 2.5% cholesterol, and 0.2% sodium cholic acid) for 4 weeks, whose blood glucose level was higher than 14 mmol/L, were randomly divided into two group (Model; *n* = 6 and JTXK-treated; *n* = 6); and as a normal control group, C57BL/6J mice (*n* = 6) were given a standard diet. JTXK-treated mice were given 1.75g/kg JTXK granule by gavage for 10 weeks each morning and evening, respectively. The normal control and diabetic groups (model) received the same amount of saline. After 10 weeks of administration, all mice were sacrificed by cervical dislocation, and the pancreas were taken out for the consecutive experiments. All the protocols were approved by the Animal Care Committee of Beijing University of Chinese Medicine, China.

### Pancreas histomorphology evaluation

Pancreas tissue was obtained from 22-week-old C57BL/6J and KKAy mice, and first fixed in 10% neutral PBS formaldehyde fixative, then embded in paraffin. Dulcimer and eosin (HE) staining was performed using sections of 4–5 μm thickness according to conventional methods. Finally, the morphological changes in islet tissue section was observed using an optical microscope (Olympus, Tokyo, Japan).

### Microarray experiment

Total RNA was isolated using TRIzol Reagent (Invitrogen) and purified using the RNeasy mini kit (QIAGEN) according to the instructions. RNA quantity and quality was determined by using nanodrop spectrophotometer (ND-1000, Nanodrop Technologies) and RNA Integrity was measured by gel electrophoresis. Total miRNAs were labeled and hybridized according to the manufacturer's (Exiqon) manual. Then the slides were scanned using the Axon GenePix 4000B microarray scanner (Axon Instruments, Foster City, CA). Scanned images were then imported into GenePix Pro 6.0 software (Axon) for grid alignment and data extraction. After median normalization, significant differentially expressed miRNAs (DEMs) between two groups were identified through Fold change and *P*-value. Finally, hierarchical clustering and volcano plot figures was completed to display distinguishable miRNA expression profile between the two groups.

### Differential miRNA target gene prediction and enrichment analysis

MirBase, Miranda, and Taregetscan, target prediction databases, were used to predict and analyze the target gene of aberrant expression of miRNA. The final result of the prediction takes the overlapping part of the gene obtained by this three databases, which were used to Gene Ontology (GO) and Kyoto Encyclopedia of Genes and Genomes (KEGG) Pathway analysis. Briefly, Fisher's exact test is used for identify GO terms and KEGG pathways which are significant correlated with differentially expressed genes (DEGs; *p* ≤0.05).

### Cell culture, transfection, and Foxo1 overexpressing model construction

INS-1 pancreatic β-cell were purchased from Institute of Basic Medical Sciences (Chinese Academy of Medical Sciences) and cultured in RPMI-1640 supplemented with 10% FBS (Gibco, Grand Island, NY, USA), 0.1% gentamicin and 0.05% biotin at 37°C under 5% CO2 sterile culture. The medium was changed every 24 h.

Lovirus LV-Foxo1 (16944-1) and negative control virus CON171 were packaged in 293T cells. INS-1 cells were infected with lentivirus for 36 h (CON), and the stable expression of Foxo1 cell (OE) line and the negative control cell line (NC) was obtained by puromycin screening. Then 2.5 μg/mL Doxycycline intervention induced Foxo1 expression. The expression of Foxo1 gene and protein was detected by qRT-PCR and Western blot. Compared with NC group, the relative expression of Foxo1 gene and protein were statistically significant, indicating that cell modeling was successful.

### Quantitative real-time PCR verification

Total RNA was extracted from pancreas tissue and INS-1-Foxo1 overexpressing model cells. Total RNA (2 μg) was converted to cDNA according to manufacturer protocol (Invitrogen Life Technologies, USA), and miRNA expression was measured by quantitative PCR using SYBR Premix ExTaq and an MX3000 instrument. The primers and genes used in this research are listed in Table 1. The PCR data were normalized to U6 and β-Actin to calculate miRNA- mmu-miR-378a-3p and Akt, respectively.

**Table 1:** MiRNA and mRNA primers for quantitative PCR analysis.

**Primer name**	**Sequence**
U6 (H)	F: 5′GCTTCGGCAGCACATATACTAAAAT3′
	R: 5′CGCTTCACGAATTTGCGTGTCAT3′
mmu-miR-378a-3p	GSP:5′GGGCACTGGACTTGGAGTC3′
	R: 5′GTGCGTGTCGTGGAGTCG3′
Akt	F: 5′-ACTCATTCCAGACCCACGAC-3′
	R: 5′-CCGGTACACCACGTTCTTCT-3′
β-Actin (H)	F: 5′-GTGGCCGAGGACTTTGATTG-3′
	R: 5′-CCTGTAACAACGCATCTCATATT-3′

*GSP: a specific primer for miRNAs*.

### Western blot

Immunoblotting for Akt, p-Akt, Foxo1, p-Foxo1, and GAPDH was performed according to the routine method. Primary antibodies from Santa Cruz Biotechnology (Santa Cruz, CA, USA) were used for INS-1 cells. After incubation with the secondary antibodies for 1.5 h, the proteins were detected using the ECL kit according to standard procedures (Thermo Fisher Scientific Inc., China) and quantified using Quantity One (Bio-Rad, USA).

### Statistical analysis

SPSS software (Version 20.0) was used in this study for the statistical analyses. The results are expressed as mean ± SEM (standard error). Comparisons between multiple groups was analyzed using one-way ANOVA analysis. Two-sided *P*-values < 0.05 were considered statistically significant. Fisher's exact test is used for identify GO terms and KEGG pathways which are significant correlated with differentially expressed genes (DEGs) (*p* ≤ 0.05).

## Results

### JTXK granule reduced the blood glucose level and body weight in KKAy diabetic mice

**Figure 2:**
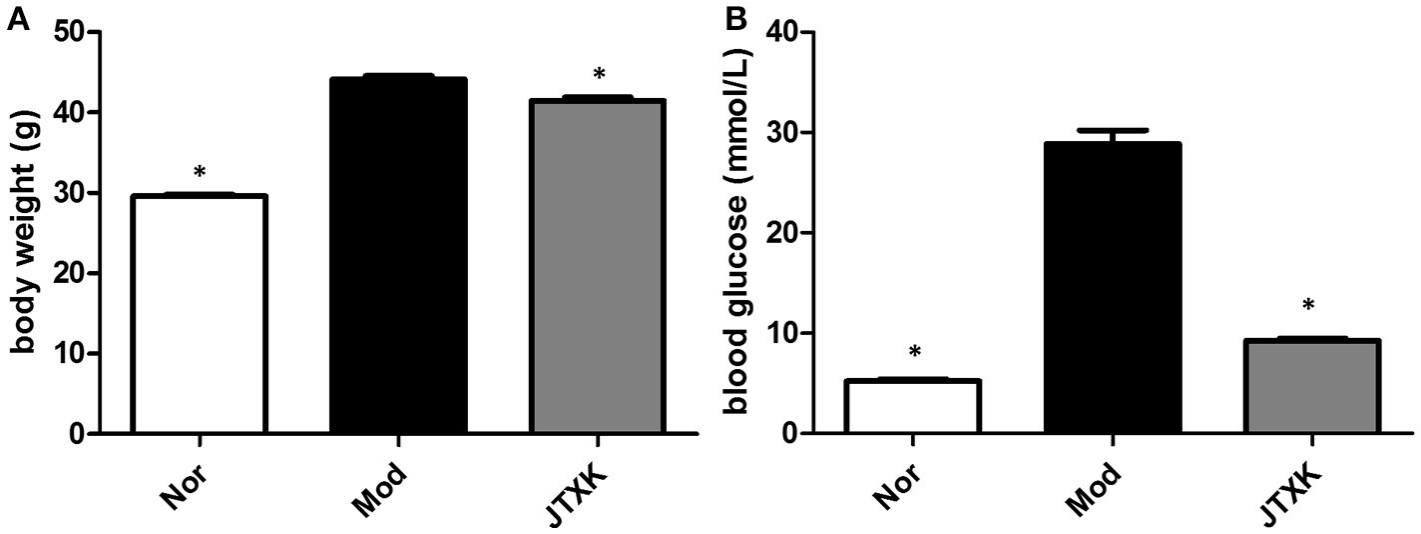
JTXK granule decreased the body weight **(A)** and the blood glucose level **(B)**. Data are expressed as mean ± SE. ^*^P < 0.05 compared with model group (KKAy diabetes mice), *N* = 6.

After 10 weeks of JTXK granule treated, the fasting blood glucose levels of each group mice were measured by a glucometer and the body weight of each group was also measured at the same time. The glycaemia and body weight in the KKAy diabetic mice group was significantly higher than the JTXK-treated group (*P* ≤ 0.01; Figure 2), suggesting that the JTXK granule could significantly reduce the blood glucose level and body weight in KKAy diabetic mice.

### JTXK granule reduced the damage of pancreas tissue in KKAy diabetic mice

**Figure 3:**
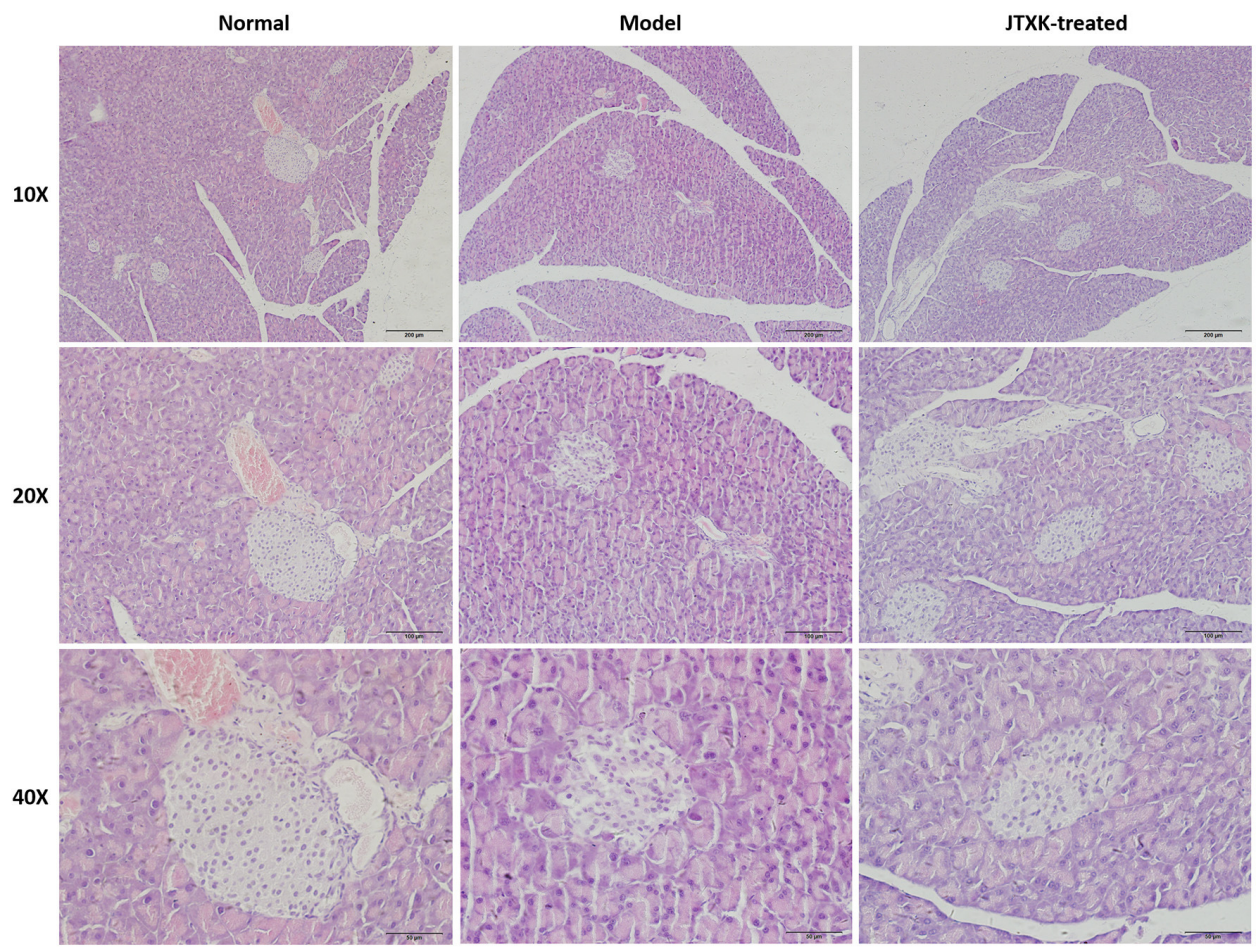
HE staining of pancreatic tissue in mice (Original magnification, 10, 20, and 40x). C57BL/6 normal control group (*n* = 6), High-fat diet induced KKAy diabetic model group (*n* = 6) and (C) JTXK granule-treated group (*n* = 6).

The pathological changes of islet cells in diabetic mice and the protective effect of JTXK granules on the pancreas tissue of diabetic mice were evaluated by HE staining (Figure 3). Histological observation found that the islets of normal mice showed typical histological structure. Compared with normal pancreas, the islet boundaries of the KKAy diabetic mice (model) were unclear, and the number of islet cells were significantly reduced and irregularly arranged. In addition, islet cells appears compensatory hypertrophy. At the same time there are exist apoptotic cells which cytoplasmic are swelling and the nuclear are pyknosis. After the JTXK granule treatment, the injury of pancreatic tissue and the apoptosishas of islet cells were both decreased, and the number of islet cells were increased significantly.

### Effects of JTXK granule on the miRNA expression in pancreas tissue of diabetic mice

**Figure 4:**
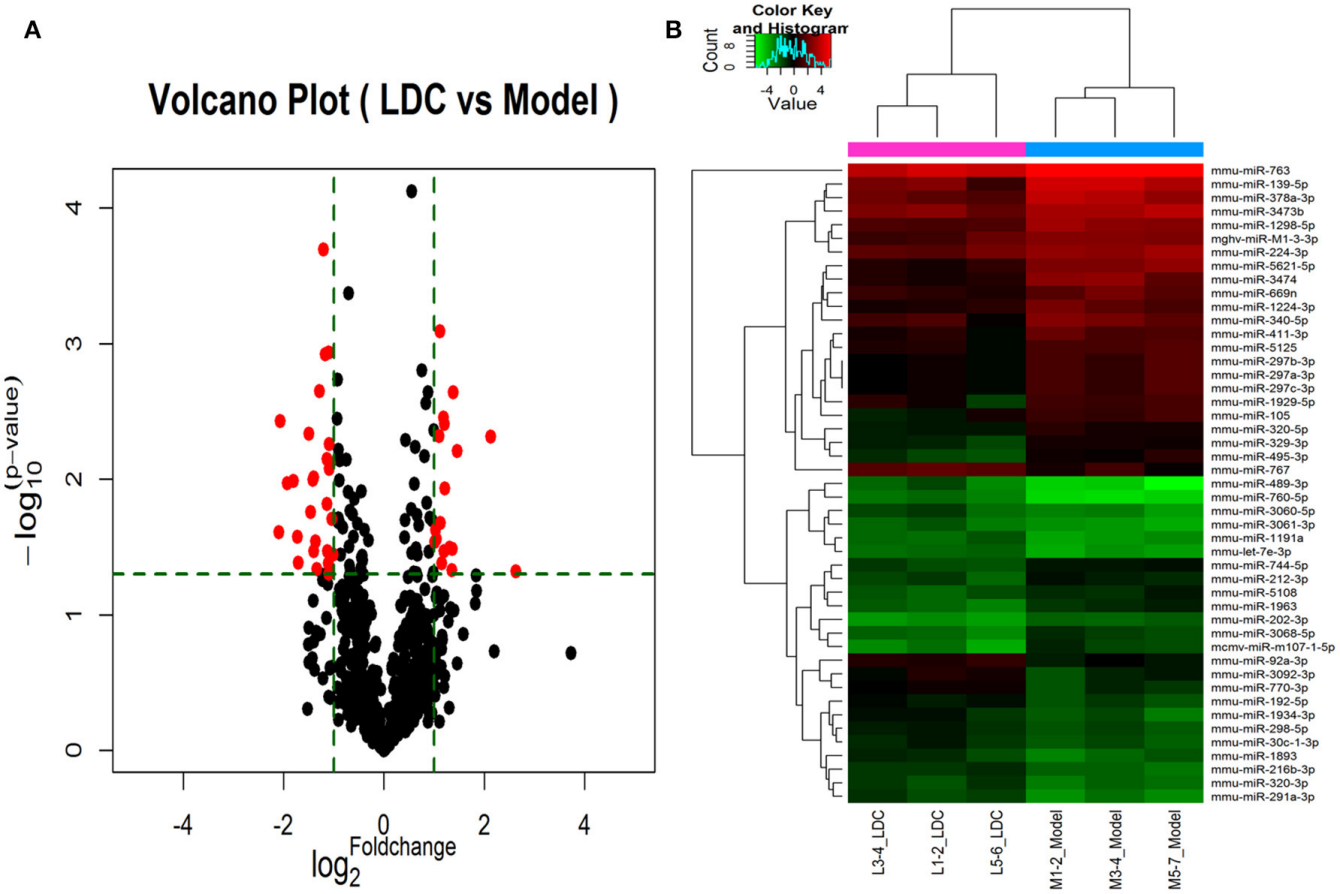
Volcano plot **(A)** and Hierarchical clustering **(B)** of miRNAs in KK-Ay diabetic group (Model) and JTXK-treated group (LDC). **(A)**Volcano plot was constructed using *p*-values and fold-change of miRNAs, with -log (*P*-value) as the ordinate and log2 (Fold change) for the abscissa. Red dots represent miRNAs that are differentially expressed between Model and LDC (*P* ≤ 0.05). **(B)** Hierarchical clustering was constructed according to the expression levels of miRNA, the six samples were classified into two groups (Model or LDC). Green represents low relative expression, and red represents high relative expression.

**Figure 5:**
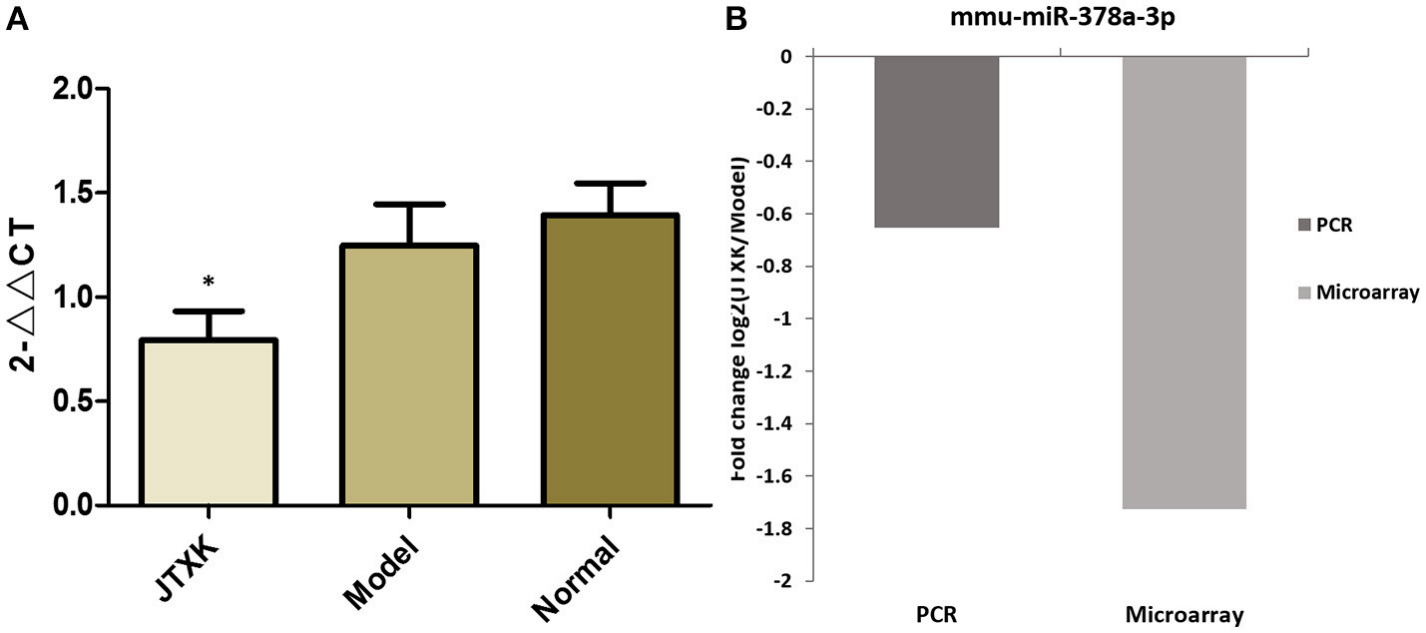
Validation of microarray data by qRT-PCR. **(A)** Comparison of mmu-miR-378a-3p qRT-PCR data between groups, **(B)** Comparison of the results of qRT-PCR and microarray for mmu-miR-378a-3p. Results obtained with these two methods were consistent with each other. ^*^P < 0.05 vs. model group (KKAy diabetic mice) and normal group (C57BL/6J mice), *N* = 6.

A total of 45 DEMs were detected from pancreas tissue of KK-Ay diabetic group and JTXK-treated group by six miRNA microarray experiments (Fold change>2 and *P* ≤ 0.05), of which 18 miRNAs are upregulated and 27 miRNAs are downregulated (Figure 4; Tables 2, 3; Supplementary Figure 1). In addition, hierarchical clustering analysis showed that the DEMs could make samples easily divided into two groups, i.e., the KKAy diabetic mice and JTXK-treated groups (Figure 4). The down-regulated expressed mmu-miR-378a-3p were selected for qRT-PCR validation, and the results showed a good consistency (Figure 5). Thence, the differences in miRNA expression between KKAy diabetic mice and JTXK Granule Treatment groups suggest that JTXK granule can alter the expression of miRNAs in the pancreas tissue of KKAy diabetic mouse.

**Table 2:** Up-regulated miRNAs between diabetic and JTXK-treated mice pancreas tissue samples.

**ID**	**Name**	**Fold change**	***P*-value**
42796	mmu-miR-489-3p	6.1532	0.0476
42741	mmu-miR-760-5p	4.3499	0.0048
42595	mmu-miR-291a-3p	2.7422	0.0062
148051	mmu-miR-770-3p	2.5975	0.0023
148487	mmu-miR-1934-3p	2.5618	0.0325
148055	mmu-miR-3060-5p	2.5477	0.0468
148104	mmu-miR-3092-3p	2.4797	0.0321
146111	mmu-miR-767	2.3098	0.0117
17732	mmu-miR-192-5p	2.3043	0.0039
148486	mmu-miR-3061-3p	2.2900	0.0339
42743	mmu-let-7e-3p	2.2716	0.0035
146071	mmu-miR-1893	2.2128	0.0416
46239	mmu-miR-1191a	2.1735	0.0211
148295	mmu-miR-216b-3p	2.1594	0.0008
169075	mmu-miR-92a-3p	2.1397	0.0048
27533	mmu-miR-320-3p	2.0652	0.0276
27572	mmu-miR-298-5p	2.0288	0.0235
42702	mmu-miR-30c-1-3p	2.0057	0.0289

**Table 3:** Down-regulated miRNAs between diabetic and JTXK-treated mice pancreas tissue samples.

**ID**	**Name**	**Fold change**	***P*-value**
148653	mmu-miR-3474	0.2337	0.0245
168835	mmu-miR-5621-5p	0.2378	0.0037
145677	mmu-miR-139-5p	0.2628	0.0106
42676	mmu-miR-495-3p	0.2852	0.0103
148668	mmu-miR-378a-3p	0.3026	0.0266
46844	mcmv-miR-m107-1-5p	0.3049	0.0410
46381	mmu-miR-1298-5p	0.3541	0.0046
	mmu-miR-297a-3p/		
42585	mmu-miR-297b-3p/	0.3635	0.0175
	mmu-miR-297c-3p		
146031	mmu-miR-1963	0.3745	0.0101
148490	mmu-miR-1224-3p	0.3790	0.0338
42851	mmu-miR-105	0.3792	0.0097
148559	mmu-miR-411-3p	0.3866	0.0287
29872	mmu-miR-340-5p	0.3957	0.0460
11227	mmu-miR-329-3p	0.4104	0.0022
27855	mmu-miR-763	0.4330	0.0002
27568	mmu-miR-744-5p	0.4420	0.0012
168828	mmu-miR-5125	0.4531	0.0071
169344	mmu-miR-3473b	0.4537	0.0152
146081	mmu-miR-1929-5p	0.4581	0.0338
148391	mmu-miR-3068-5p	0.4626	0.0415
11207	mmu-miR-202-3p	0.4644	0.0012
146050	mmu-miR-669n	0.4658	0.0494
17537	mghv-miR-M1-3-3p	0.4667	0.0055
146163	mmu-miR-224-3p	0.4674	0.0084
147366	mmu-miR-320-5p	0.4773	0.0076
169248	mmu-miR-5108	0.4862	0.0196
42627	mmu-miR-212-3p	0.4931	0.0358

### Differentially expressed miRNAs target prediction and network analysis

**Figure 6:**
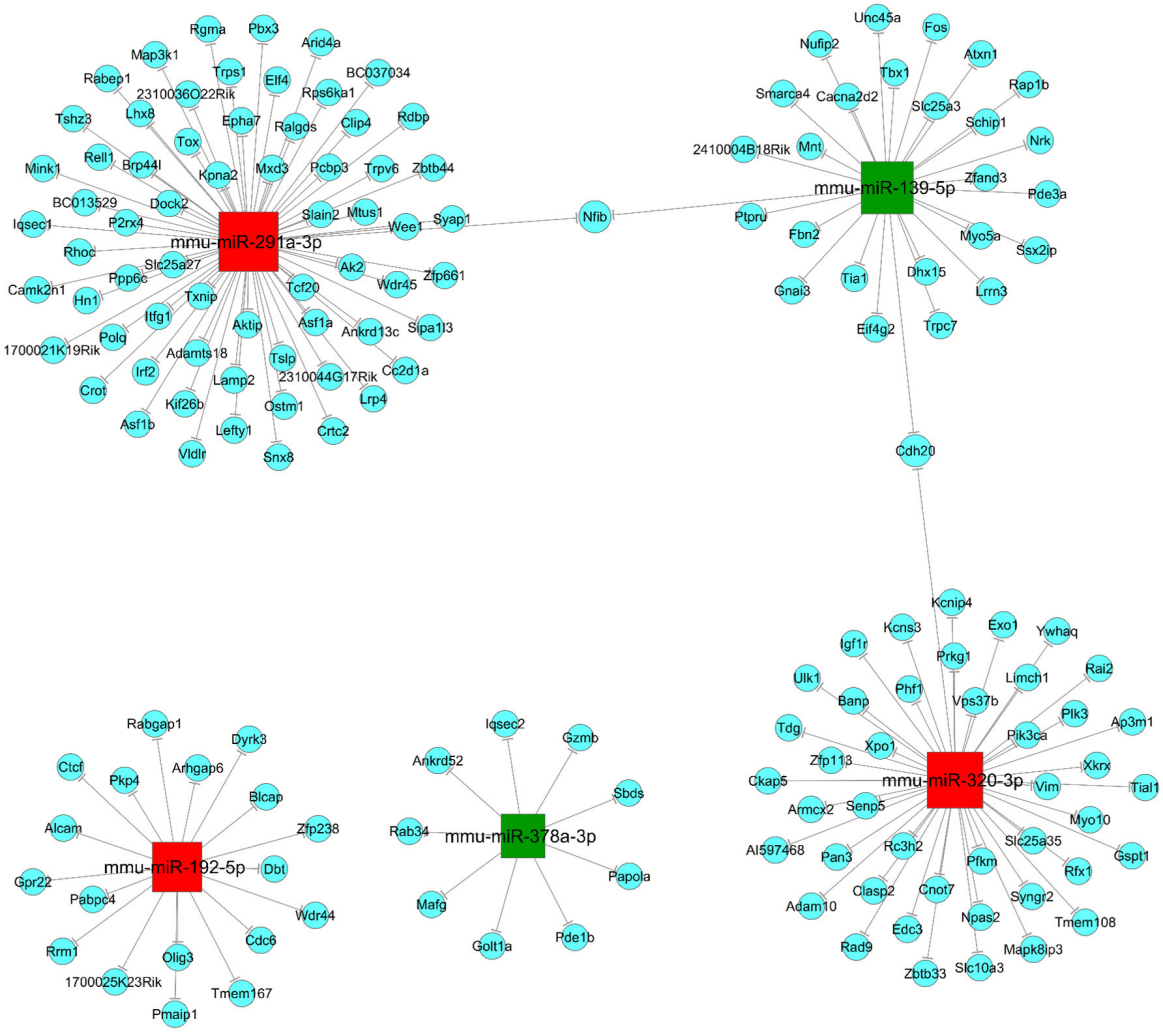
miRNA-mRNA network. Red (mmu-miR-192-5p, mmu-miR-291a-3p and mmu-miR-320-3p) and Green (mmu-miR-139-5p and mmu-miR-378a-3p) are square nodes represent up-regulated and down-regulated miRNAs, respectively. Blue round nodes are represent mRNAs and the solid line between the two nodes represents a correlation.

Three hundred and eight up-regulated mRNAs and 326 down-regulated mRNAs were predicted from the DEMs by overlapping three target prediction databases (mirbase, Miranda, and mirdb; Appendix 1 in Supplementary Materials). It has been shown that a single miRNA has the ability to target several groups of genes within a signaling pathway, and multiple miRNAs can build up as a network to collectively target a key gene in a signaling pathway (Sud et al., 2017). Therefore, to understand how JTXK granules can alter the expression of miRNAs and their target mRNA expression changes are key to understanding the molecular mechanisms of its anti-diabetic effects. In this study, we selected five DEMs, three upregulated (mmu-miR-192-5p, mmu-miR-291a-3p and mmu-miR-320-3p), and two downregulated (mmu-miR-139-5p and mmu-miR-378a-3p), to construct the miRNA-mRNA network (Figure 6). According to the network we can intuitive to see that, mmu-miR-192-5p was associated with 18 mRNAs, mmu-miR-291a-3p was associated with 66 mRNAs and mmu-miR-320-3p was associated with 44 mRNAs. Furthermore, the downregulated miRNAs mmu-miR-139-5p and mmu-miR-378a-3p were associated with 27 mRNAs and 9 mRNAs, respectively.

### GO enrichment analysis

**Figure 7:**
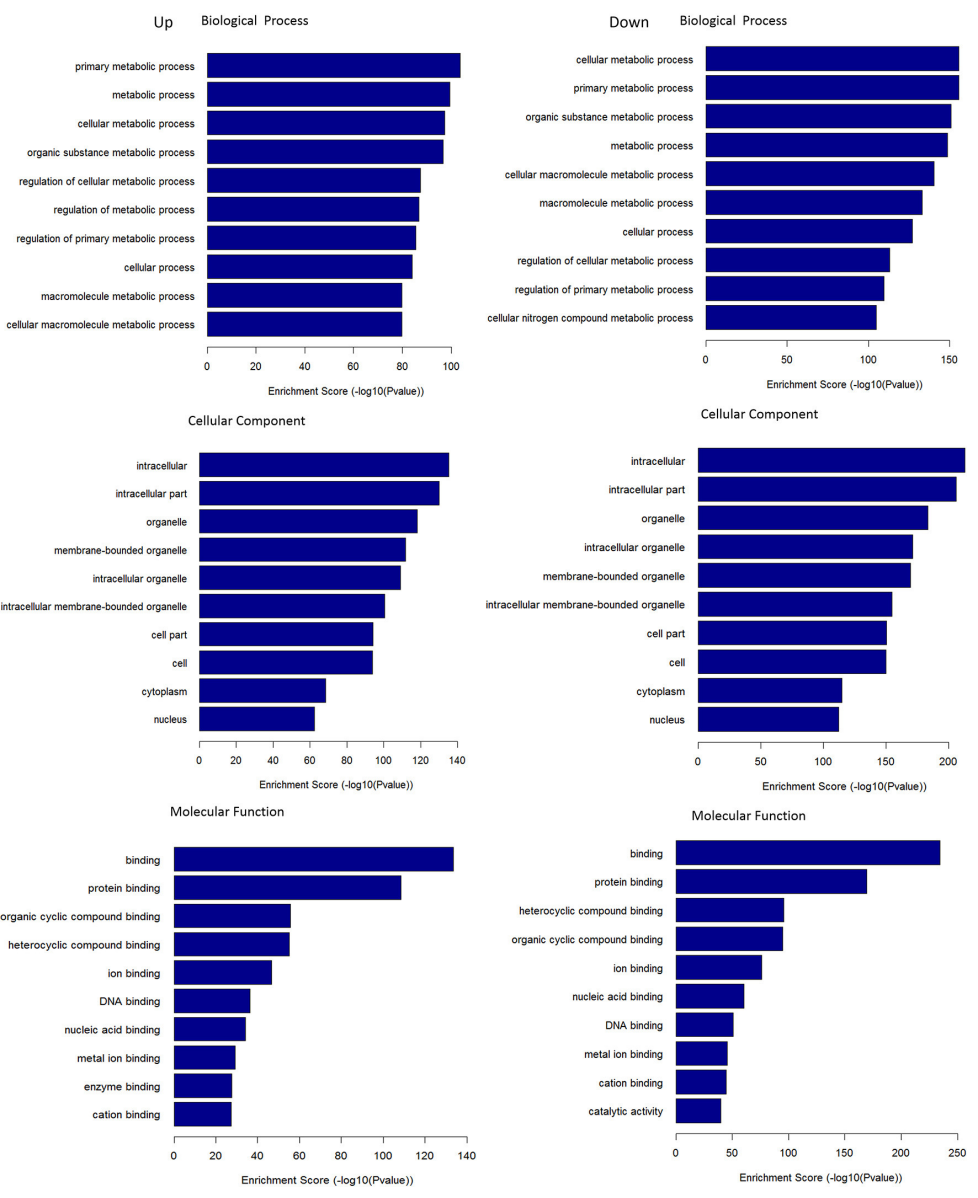
GO terms for DEMs. GO enrichment score [–log 10 (*P*-value)] analysis of up-regulated mRNAs and down-regulated mRNAs with top 10.

Through the GO enrichment analysis of the DEGs, we can find different entries that enriched in Biological Process (BP), Cellular Component (CC), and Molecular Function (MF) of the DEGs in pancreas tissue from KK-Ay diabetic mice and JTXK Granule-treated groups. Up-regulated mRNAs were mainly enriched in metabolic process (BP), intracellular (CC), and binding (MF). On the other hand, the down-regulated mRNAs were enriched in cellular metabolic process (BP), intracellular (CC), and binding (MF). Here, we according to the enrichment factor listed the top ten GO terms including up-regulation and down-regulation mRNAs (Figure 7).

### KEGG pathway analysis

**Figure 8:**
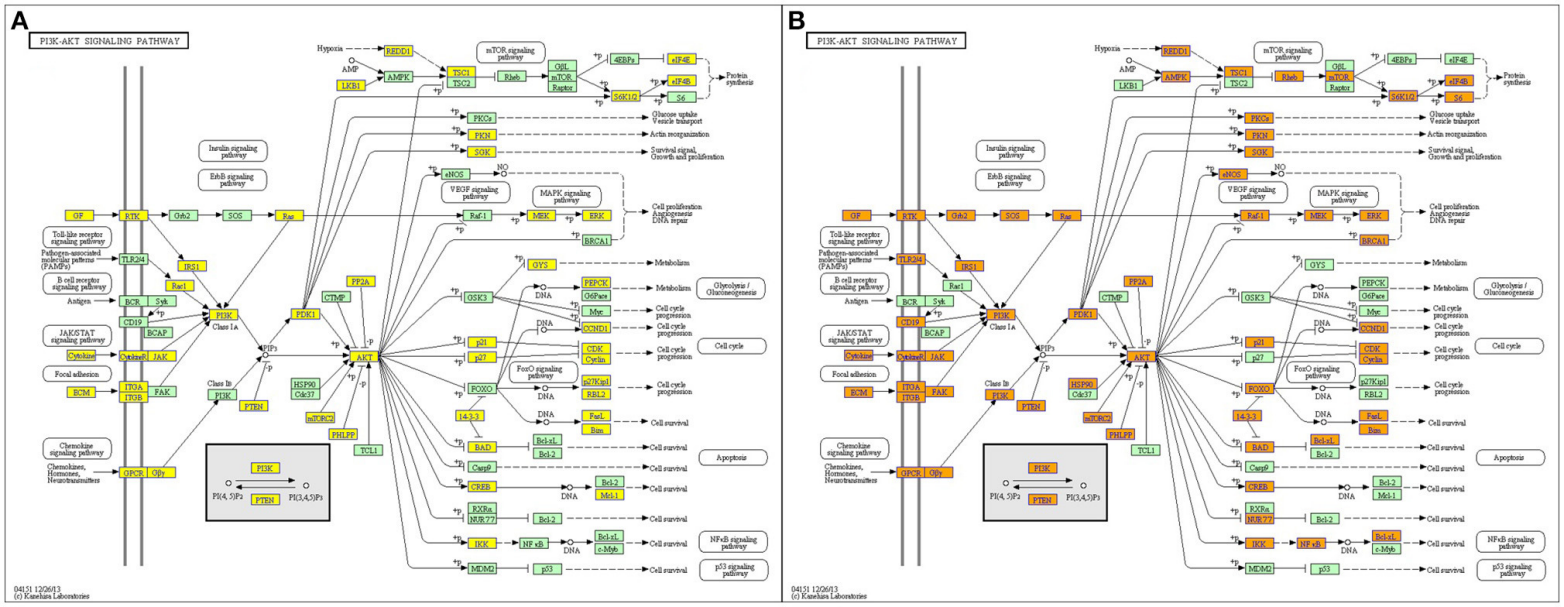
PI3K-Akt signaling pathway. **(A)** Up and **(B)** Down, Orange marked nodes are associated with up-regulated or only whole dataset genes, yellow marked nodes are associated with down-regulated genes, green nodes have no significance.

KEGG pathways enrichment analysis showed that the up-regulated mRNAs and the down-regulated mRNAs in the JTXK Granule-Treated groups were participated in 94 and 113 pathways, respectively. Which up-regulated mRNAs mainly enrichment in PI3K-Akt, Foxo, Regulation of actin cytoskeleton and Insulin signaling pathway, and the down-regulated mRNAs were mainly participated in MAPK, PI3K-Akt, Wnt, Hippo, and Insulin signaling pathway (Table 4). Pathway analysis revealed that PI3K-Akt signaling pathway (Figure 8) and others metabolic-related pathways (e.g., FoxO and MAPK signaling pathway) are closely associated with the DEMs.

**Table 4:** KEGG pathways of differentially expressed mRNAs between diabetic and JTXK-treated mice islet tissue samples.

**Pathway ID**	**Definition**	**FDR**	**Enrichment score**
**UP-REGULATED**			
mmu04151	PI3K-Akt signaling pathway	2.1E-13	15.12
mmu04510	Focal adhesion	2.68E-11	12.71
mmu04068	FoxO signaling pathway	4.27E-10	11.33
mmu05215	Prostate cancer	1.99E-08	9.54
mmu05210	Colorectal cancer	3.99E-07	8.14
mmu04810	Regulation of actin cytoskeleton	1.49E-06	7.49
mmu05212	Pancreatic cancer	3.01E-06	7.12
mmu05161	Hepatitis B	3.52E-06	6.99
mmu05200	Pathways in cancer	4.21E-06	6.86
mmu04150	mTOR signaling pathway	5.49E-06	6.70
**DOWN-REGULATED**			
mmu05200	Pathways in cancer	1.99E-12	14.14
mmu04010	MAPK signaling pathway	4.81E-09	10.46
mmu04151	PI3K-Akt signaling pathway	7.16E-09	9.99
mmu04510	Focal adhesion	7.16E-09	9.96
mmu04660	T cell receptor signaling pathway	7.16E-09	9.89
mmu05205	Proteoglycans in cancer	9.8E-08	8.67
mmu05215	Prostate cancer	9.8E-08	8.61
mmu04310	Wnt signaling pathway	1.14E-06	7.48
mmu04068	FoxO signaling pathway	1.45E-06	7.30
mmu05214	Glioma	1.45E-06	7.28

### Effects of JTXK granule on the significant mRNA and protein expression in the PI3K/Akt/FoxO signaling pathway in INS-1 pancreatic β-cell

**Figure 9:**
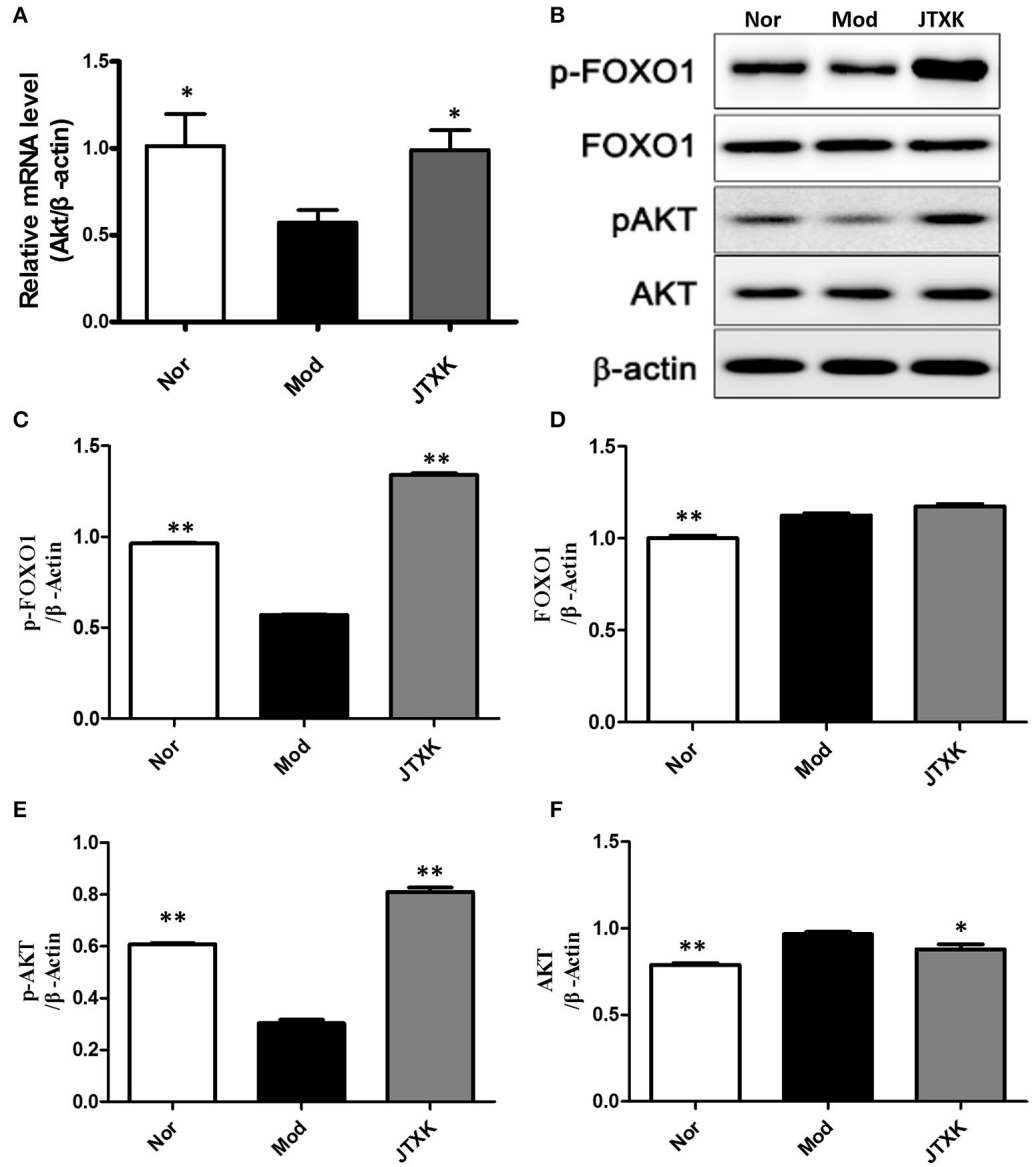
JTXK granule influenced relevant mRNA **(A)** and protein expression **(B–F)** in the PI3K/Akt signaling pathway in INS-1 cell. Akt, serine/threonine-protein kinase; p-Akt, phosphorylated Akt. Nor (Normal Cell + Normal mouse serum); Mod (Model Cell + Normal mouse serum); JTXK (Model Cell + 10% Contains JTXK Granules Serum). Data are expressed as the mean ± SE of four independent experiments. ^*^P < 0.05, ^**^P < 0.01 vs. model group (KKAy diabetes mice).

As shown in Figure 9A, the mRNA expression of Akt in the model group was significantly lower than those in the control group (*P* < 0.05) and the JTXK granule can increased the mRNA expression of Akt (*P* < 0.05). The expression of the proteins Akt, p-Akt, p-Akt/Akt, and p-Foxo1 were also significantly increased in the JTXK granule treatment group (Figures 9B–F), and this finding was consistent with the mRNA expression in the various groups. The results show that JTXK granule may help control the blood glucose of diabetic patients by activating PI3K signaling pathway in INS-1 islet β cells.

## Discussion

Type 2 diabetes is an endocrine and metabolic disorders caused by the combination of genetic susceptibility and environmental factors and could be characterized by nutrient metabolic disorders. The damage of β-cell is an important basis pathological of diabetes. Our previous study found that JTXK granule have a good effect on protecting the function of β-cells, promoting insulin secretion, and increasing the sensitivity of tissue to insulin (Zhao et al., 2014; Na et al., 2016; Rui et al., 2016; Zhang et al., 2016a,b). In this study, we found that JTXK granule have a good protective effect on pancreatic tissue in KKAy diabetic mice induced by HFD. In order to further study its pharmacological mechanism, we used miRNA microarray to explore the regulation of JTXK granule on the level of miRNA in pancreatic tissue of diabetic mice.

In the current study, a total of 1174 miRNAs were detected. Among them, there are 18 up-regulated and 27 down-regulated miRNAs with significant differences in the pancreatic histological of the JTXK granule treated group. A single miRNA has the potential to regulate multiple mRNAs, and a single mRNA can be regulated by multiple miRNAs, which enables them to regulate several groups of mRNAs within a network or signaling pathway and have a strong impact on diverse cellular processes (Sud et al., 2017). Studies have shown that miRNAs and their target genes are involved in the pathogenesis of type 2 diabetes (Distefano, 2016). More importantly, miRNAs can play a key role in the pathogenesis of metabolic diseases by influencing the status and function of the pancreas to regulate lipid and glucose metabolism (Iacomino and Siani, 2017). Thus, understanding how JTXK granule can alter the expression of miRNAs and their target mRNA in pancreatic tissue is key to understanding the molecular mechanism of JTXK granule in anti-diabetic action. Therefore, we selected five DEMs, three upregulated (mmu-miR-192-5p, mmu-miR-291a-3p and mmu-miR-320-3p), and two downregulated (mmu-miR-139-5p and mmu-miR-378a-3p), construct miRNA-mRNA network to explore the regulatory mechanisms of miRNAs.

It has been found that miR-192-5p related to apoptosis and tyrosine kinase signaling pathway (Wu et al., 2015), and the reduction of miR-192-5p expression usually occurs in the more severe stages of diabetes (Ma et al., 2016). In this study, JTXK granule increased the expression of miR-192-5p in pancreatic tissue of KKAy diabetic mice, which may be a mechanism for its anti-diabetic effect. At the same time, we found that JTXK granules can reduce the expression of miRNA-139-5p in the pancreas of diabetic mice. It has been found that a decrease in miR-139-5p expression contributes to the anti-apoptotic effect on pancreatic and INS-1 cells in diabetic rats (Li et al., 2017). Since the target gene Fos of miR-139-5p can down-regulate the MAPK signaling pathway, we speculated that JTXK granules could exert anti-diabetic effects by reducing the expression of miR-139-5p and inhibiting the expression of target gene-Fos and improving MAPK signaling pathway. In addition, miR-139-5p can decrease the p-PI3K (p85) and p-Akt, which affects the normal function of IRS1/PI3K/Akt insulin signaling pathway (Mi et al., 2015). In this study, JTXK granule reduced the expression of miR-139-5p in pancreatic tissue of diabetic mice, and increased the expression of p-PI3K and p-Akt in INS-1 Foxo1 overexpressing cell. Therefore, through the microarray and cell experiments we speculate that JTXK granules can reduce the expression of miR-139-5p in pancreatic tissue of diabetic mice to promote the phosphorylation of PI3K and AKT, and then play the role of anti-diabetic.

In the miRNA and mRNA network diagram, another down-regulated miRNA miR-378a-3p, which verified by PCR and have a good consistency with the microarray results has been shown to function in regulating skeletal muscle growth and promoting the differentiation of myoblasts (Wei et al., 2016). Meanwhile, miR-378a plays an important role in adipogenesis and obesity, which can promote the adipogenesis of 3T3-L1 cells by targeting MAPK1 (Huang et al., 2015). Another study found that miR-139-5p was significantly upregulated in liver fat of mice fed with the high-fructose compared to mice fed with a normal diet (Sud et al., 2017). In present study, we found that the expression level of miR-139-5p was upregulated in pancreatic tissue of KKAy diabetic mice induced by high fat diet (HFD), whereas the expression of miR-139-5p was downregulated in the JTXK granule treatment group. From the above result, we speculate that the hypoglycemic effect of JTXK granule on reducing abnormal glucose and lipid metabolism associated with diabetes may be implemented by down-regulating the expression of miR-139-5p.

Pathway analysis showed that the DEMs in pancreas tissue between JTXK granule-treated and KKAy diabetic mice group were closely related to PI3K-Akt and FoxO signaling pathway. The miRNA-mRNA network revealed that the potential target genes of Crtc2 (miR-291a-3p), Pik3 (miR-320-3p), and Pik3ca (miR-320-3p) are associated with PI3K-Akt and FoxO signaling pathway. Furthermore, we found that JTXK granule could exert anti-diabetic effects by regulating the key proteins in the PI3K-AKT pathway, such as, PI3K, AKT, and Foxo1, in INS-1 Foxo1 overexpressing cell experiments.

Foxo1 protein is a downstream transcription factor activated by PI3K-Akt pathway, and its transcriptional activity is regulated by phosphorylation of Akt (Birkenkamp and Coffer, 2003). Akt can directly make Foxo1 phosphorylate, Phosphorylated FOX1 can be transferred from the nucleus to the cytoplasm and then lose activity (Barthel et al., 2005). Epidemiological statistics show that the polymorphism of Akt gene is closely related to diabetes mellitus, it can be activated by phosphorylation then play a role of anti-β cell apoptosis and promote the survival of β cells (Blaabjerg et al., 2014; Yin et al., 2017). Our observations suggest that high fat intake in KKAy mice can promote Foxo1 protein expression. At the same time, JTXK granules can increase Akt, p-Akt and p-Foxo1 protein expression, reduce Foxo1 expression in INS-1-Foxo1 overexpressing cells. In addition, the expression of AKT in the JTXK granule-treated group was also increased at the mRNA level. This result suggests that JTXK granule can promote the phosphorylation of Foxo1 by increasing the expression of AKT in pancreas tissue and exerting anti-diabetic effects via the PI3K/Akt/FoxO signaling pathway.

In conclusion, our research determined a novel set of miRNAs that were abnormally expressed in the pancreas of KKAy diabetic mice induced by HFD. At the same time, we found that JTXK granule acting on the regulatory network assembled by different expression miRNAs in pancreas tissue via the PI3K/Akt signaling pathway, and improve the cellular function of INS-1-Foxo1 overexpression cell model by regulating the expression of important proteins on PI3K pathway such as, PI3K, Akt, and Foxo1. This research provides a new insight into the molecular mechanism of JTXK granule in anti-diabetes effects and provides miRNAs targets for pharmaceutical design in the prevention and treatment of diabetes mellitus.

## Ethics statement

The study was conducted in strict accordance with the recommendations in the Guide for the Care and Use of Laboratory Animals from the Association for the Committee for Animal Experiments of the National Centre. All animal operations in this study were approved by the Ethics Committee of Beijing University of Chinese Medicine.

All animal operations in this study were conducted according to the guidelines of the Animal Management Committee of Beijing University of Chinese Medicine.

## Author contributions

SG and GJ designed the experiments; TA and FM wrote the manuscript; YP, HL, JM, and ZZ performed the experiments; YL, DDZ, XY, and DWZ analyzed the data. All authors reviewed the manuscript.

### Conflict of interest statement

The authors declare that the research was conducted in the absence of any commercial or financial relationships that could be construed as a potential conflict of interest.

## Supplementary material

Supplementary Figure 1 Vennplot of the miRNAs target genes. The amounts of **(A)** up-regulated and **(B)** down-regulated miRNAs target genes were overlapped in mirbase, miranda and mirdb databases.
