# Supplementary figures and images for: Jiang Tang Xiao Ke Granule Play an Anti-diabetic Role in Diabetic Mice Pancreatic Tissue by Regulating the mRNAs and MicroRNAs Associated with PI3K-Akt Signaling Pathway
Source: Front Pharmacol. 2017 Nov 1;8:795. doi: 10.3389/fphar.2017.00795 (PMC5671979; doi:10.3389/fphar.2017.00795)

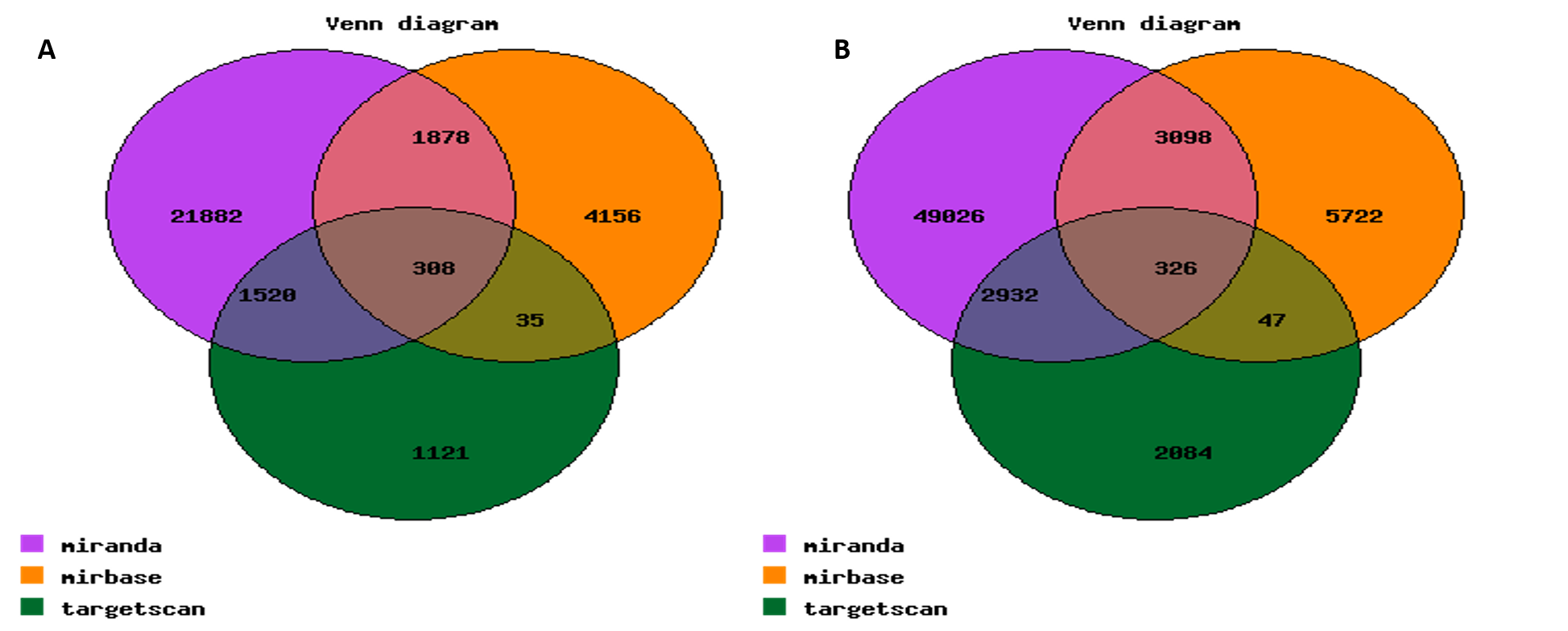

Supplement: Supplementary Figure 1 — Vennplot of the miRNAs target genes. The amounts of (A) up-regulated and (B) down-regulated miRNAs target genes were overlapped in mirbase, miranda and mirdb databases. [file Image1.TIF]
